# Glycolipids in Parkinson's disease: beyond neuronal function

**DOI:** 10.1002/2211-5463.13651

**Published:** 2023-06-04

**Authors:** Fokion Spanos, Michela Deleidi

**Affiliations:** ^1^ Institut Imagine, INSERM UMR1163 Paris Cité University France; ^2^ Aligning Science Across Parkinson's (ASAP) Collaborative Research Network Chevy Chase MD USA; ^3^ Department of Neurodegenerative Diseases, Center of Neurology, Hertie Institute for Clinical Brain Research University of Tübingen Germany

**Keywords:** central nervous system, glycolipids, immune system, inflammation, intestine, Parkinson's

## Abstract

Glycolipid balance is key to normal body function, and its alteration can lead to a variety of diseases involving multiple organs and tissues. Glycolipid disturbances are also involved in Parkinson's disease (PD) pathogenesis and aging. Increasing evidence suggests that glycolipids affect cellular functions beyond the brain, including the peripheral immune system, intestinal barrier, and immunity. Hence, the interplay between aging, genetic predisposition, and environmental exposures could initiate systemic and local glycolipid changes that lead to inflammatory reactions and neuronal dysfunction. In this review, we discuss recent advances in the link between glycolipid metabolism and immune function and how these metabolic changes can exacerbate immunological contributions to neurodegenerative diseases, with a focus on PD. Further understanding of the cellular and molecular mechanisms that control glycolipid pathways and their impact on both peripheral tissues and the brain will help unravel how glycolipids shape immune and nervous system communication and the development of novel drugs to prevent PD and promote healthy aging.

## Graphical abstract

Glycolipids are fundamental molecules for body function and changes in their levels can lead to various disorders including Parkinson's disease. Evidently, alterations in glycolipids affect multiple tissues, from central nervous system to periphery. Herein, we discuss recent findings in glycolipid dyshomeostasis with regard to neurodegeneration, focusing on Parkinson's disease, and related immune dysfunction.

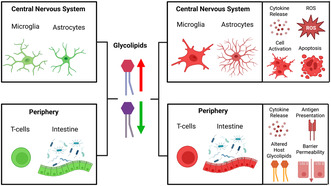

**Abbreviations**
AAV: adeno‐associated virus
AD: Alzheimer's disease
aGalCer: α‐galactosylceramide
aSMase: acidic sphingomyelinase
BBB: blood–brain barrier
bFGF: basic fibroblast growth factor
BFT: *Bacteroidetes fragilis* toxin
bSMase: bacterial sphingomyelinase
C5a: complement‐5a
C5aRa: C5aR1 antagonist
CBE: conduritol‐B‐epoxide
CNS: central nervous system
CSF: cerebrospinal fluid
DA: dopaminergic
DC: dendritic cell
EAE: experimental autoimmune encephalomyelitis
ER: endoplasmic reticulum
FTY720: fingolimod
GCase: glucocerebrosidase
GCS: glucosylceramide synthase
GD: Gaucher disease
GF: germ‐free
GI: gastrointestinal
GL: glycolipid
IBD: inflammatory bowel disease
iNKT: invariant natural killer T cells
iNOS: inducible nitric oxide synthase
LacCer: lactosylceramide
LPS: lipopolysaccharide
LSD: lysosomal storage disorder
MDC: monodansylcadaverine
MINCLE: macrophage‐inducible C‐type lectin
MoDC: monocyte‐derived dendritic cell
MPP: 1‐methyl‐4‐phenylpyridinium
MPTP: 1‐methyl‐4‐phenyl‐1,2,3,6‐tetrahydropyridine
MS: multiple sclerosis
NBDNJ: *N*‐butyldeoxynojirimycin
NPC: Niemann‐Pick disease type C
nsGL: neolacto‐series glycolipid
PD: Parkinson's disease
PDOP: d‐threo‐1‐phenyl‐2‐decaanoylamino‐3‐morpholino‐1‐propanol
S1P: sphingosine‐1‐phosphate
S1PR: sphingosine‐1‐phosphate receptor
SNpc: substantia nigra pars compacta
SPHK1: sphingosine kinase 1
SPT: serine palmitoyl transferase
TLR4: toll‐like receptor 4
UGCG: UDP‐glucose ceramide glucosyltransferase
WT: wild‐type
β‐GlcCer: glucosylceramide

## Introduction

Parkinson's disease (PD) is the most common age‐related neurodegenerative movement disorder [1]. The pathological hallmark of PD is the loss of dopaminergic (DA) neurons in the substantia nigra pars compacta (SNpc) [1]. Clinically, PD is characterized by a variety of motor symptoms mostly caused by the loss of DA neurons, including bradykinesia, muscular rigidity, tremor at rest, and postural instability [1]. The involvement of neuronal populations and brain regions outside the SN and in the peripheral nervous system contributes to the development and progression of a variety of other motor symptoms, which are poorly responsive to l‐dopa, and debilitating nonmotor complications (psychosis, cognitive impairment and dementia, depression, autonomic dysfunction, and sleep disorders) [2, 3]. Interestingly, the majority of PD patients experience nonmotor symptoms involving the gastrointestinal (GI) tract, such as chronic constipation, several years before the onset of motor symptoms [4]. The great majority of PD cases are sporadic, but the interaction between genetic susceptibility and environmental factors is generally thought to contribute to the disease [5]. However, studies of mutations in genes linked to inherited forms of PD provide several clues that are key to understanding disease pathogenesis [6]. Furthermore, the identification of genes linked to PD susceptibility is crucial for understanding mechanisms of neuronal death in both inherited and sporadic forms of the disease. Among these genes, mutations in the *GBA1* gene, which encodes the lysosomal hydrolase glucocerebrosidase (GCase), have been identified as the most common risk factor for sporadic PD and other synucleinopathies [7, 8]. GCase catalyzes the hydrolysis of glucosylceramide (β‐GlcCer), a membrane glycolipid (GL), to ceramide and glucose [9]. Biallelic *GBA1* mutations lead to Gaucher disease (GD), the most common lysosomal storage disease characterized by decreased GCase activity and the subsequent accumulation of β‐GlcCer and glucosylsphingosine in several organs, including the brain [10]. In recent years, GD and PD have been connected based on the clinical observation of parkinsonism and Lewy body pathology in patients with GD [11, 12]. Compared with the general population, patients with GD type 1 have a 20‐fold increased lifetime risk of developing parkinsonism [13], whereas individuals carrying heterozygous *GBA1* mutations have a five times greater risk of developing PD than noncarrier individuals [8, 14, 15]. Interestingly, sporadic PD patients display decreased GCase activity even in the absence of *GBA1* mutations [16], suggesting a role for GCase function and GLs in sporadic PD development. The pathological mechanisms through which the mutant enzyme causes PD remain elusive, and both gain‐ and loss‐of‐function theories have been postulated [17, 18, 19, 20, 21]. Interestingly, other lysosomal storage disorders (LSDs) displaying GL alterations are linked to an increased risk for PD, including Niemann–Pick disease type C (NPC) and Batten disease (also known as neuronal ceroid lipofuscinosis, NCL) [22, 23, 24]. These conditions are all characterized by immune changes in both the periphery and the central nervous system (CNS) [25, 26, 27, 28]. While brain damage and DA neuron dysfunction have been a research focus for many years, the role of other cell types should also be considered. Within the CNS, ceramides and GLs modulate glial cell activation [29, 30, 31, 32, 33] and blood–brain barrier (BBB) permeability [34, 35]. Outside the CNS, GLs can affect immune cell responses at different levels [36, 37, 38], including the response of intestinal cells to bacteria and bacterial metabolism [39, 40, 41, 42]. In this review, we discuss recent advances in the link between GL metabolism and immune function and how these metabolic changes may exacerbate immunological contributions to neurodegenerative diseases, with a focus on PD.

## Glycolipids

Glycolipids are a subclass of lipids found in the cell membranes of organisms from bacteria to humans [43] and consist of a hydrophobic ceramide backbone linked to a hydrophilic glycan head group [34]. GLs differ based on the type of carbohydrate and the chain length, saturation, and hydroxylation levels of ceramides [44]. GL biosynthesis involves several pathways and enzymes. The *de novo* pathway begins in the endoplasmic reticulum (ER) with the generation of ceramide [45]. A glucose moiety is then transferred to ceramide, generating β‐GlcCer at the early Golgi [45]. Further processing involves enzymatic reactions within the early (neutral GLs) or late Golgi (gangliosides) [44]. β‐GlcCer is converted into lactosylceramide (LacCer), which can then form GA2, GM3, GB3, and LC3 [34, 45]. These molecules can then be used to form GLs in the o‐, a‐, b‐ and c‐series (for GA2 and GM3), globo‐series (for GB3), and lacto(neo)‐series (for LC3) [34, 45]. The roles of GLs are diverse and include cell signaling, survival, migration, transport, inflammation, and cell death [44].

## Glycolipids in the central nervous system in health and disease

Glycolipids are highly abundant in the CNS [46], where they are mostly represented by GM1a, GD1a, GD1b, and GT1b. They all consist of two galactose, one *N*‐acetyl‐d‐galactosamine and one glucose moiety attached to the ceramide, and they differ with respect to the number and location of *N*‐acetylneuraminic acids [45, 47]. The tissue GL content depends on the brain region and cell type [48]. Gray matter mostly contains gangliosides, whereas white matter is enriched in galactosylceramides [48]. Furthermore, the brain GL content undergoes changes throughout development and aging [49, 50]. GLs play different roles in the CNS, including cell differentiation, cell–cell interactions, dendritic development, and synaptic formation, as well as transmission and energy metabolism [51, 52, 53, 54, 55, 56, 57].

Changes in GL metabolism and levels are observed in PD with aging [45, 58]. For instance, the total levels of GLs increase with age in the SN of PD patients but not in healthy controls [59, 60]. Such a change is largely attributed to the increase in β‐GlcCer levels. In contrast, ganglioside levels (GM1a, GD1a, GD1b, and GT1b) are reduced overall in the SN of PD patients compared with controls [59, 60]. Sex‐related changes are also present, as male PD patients showed reduced GD1a and GD1b levels and increased sphingomyelin in the SN compared with controls in a small cohort study. Interestingly, female PD patients did not display such differences [61]. Furthermore, a reduction in long‐chain ceramides is observed in the anterior cingulate cortex, but not in the occipital cortex, of PD patients [62]. GL changes are also observed in the cerebrospinal fluid (CSF) and serum of PD patients [59]. LacCer and GM3 are increased, whereas GM2, GD3, GD1a, GD1b, and GT1b are reduced in the CSF of PD patients [59]. Serum GM1a and GD1a are reduced in PD patients [59]. Interestingly, serum ganglioside levels are significantly reduced in patients diagnosed with rapid eye movement sleep behavior disorder, who are at risk of developing PD [59]. As gangliosides are critical for CNS function, they have been extensively studied in health and disease [63]. Interestingly, GM1, among its different neuroprotective functions, can also interact with alpha‐synuclein (α‐syn) to maintain it in its helical form [64]. The neuronal‐specific role of gangliosides in PD has already been reviewed in detail elsewhere [63, 65, 66]. In this review, we will mostly focus on the role of gangliosides in non‐neuronal cells.

Interestingly, GLs may be involved in other diseases that are directly or indirectly linked to PD. For instance, GLs have been identified as key players and immune therapeutic targets in inflammatory bowel disease (IBD) [67]. Inflammation in the intestinal tract can be caused by an imbalance in the ceramide pathway [39, 67]. Interestingly, ceramide and its related lipids, such as sphingosine‐1‐phosphate (S1P), which acts as a signaling molecule, may also play a protective role in IBD [67]. Given the potential role of gut pathology as an initiating factor of PD [68], gut‐related GLs could potentially play a role in disease onset and progression. Interestingly, the ceramide pathway is involved in melanoma [69, 70]. PD patients have a reduced risk of developing many types of cancer [71] except melanoma [72], whereas melanoma patients have an increased risk for developing PD [72, 73]. From a genetic point of view, mutations in PD‐associated genes (*SCNA*, *LRRK2*, *PPKN*, and *PINK1*) are linked to melanoma [74]. At the same time, the melanoma‐associated gene *GPNMB*, which encodes a glycoprotein involved in a variety of cancers, is elevated in PD patients and conduritol‐B‐epoxide (CBE)‐treated mouse models [31, 75]. Additionally, GPNMB inhibits the inflammatory response of astrocytes by binding to CD44R [76]. Last, GPNMB correlates with disease severity in GD patients who have an increased risk of developing certain cancers, including melanoma [77].

## Glycolipids beyond neuronal cells

**Fig. 1:**
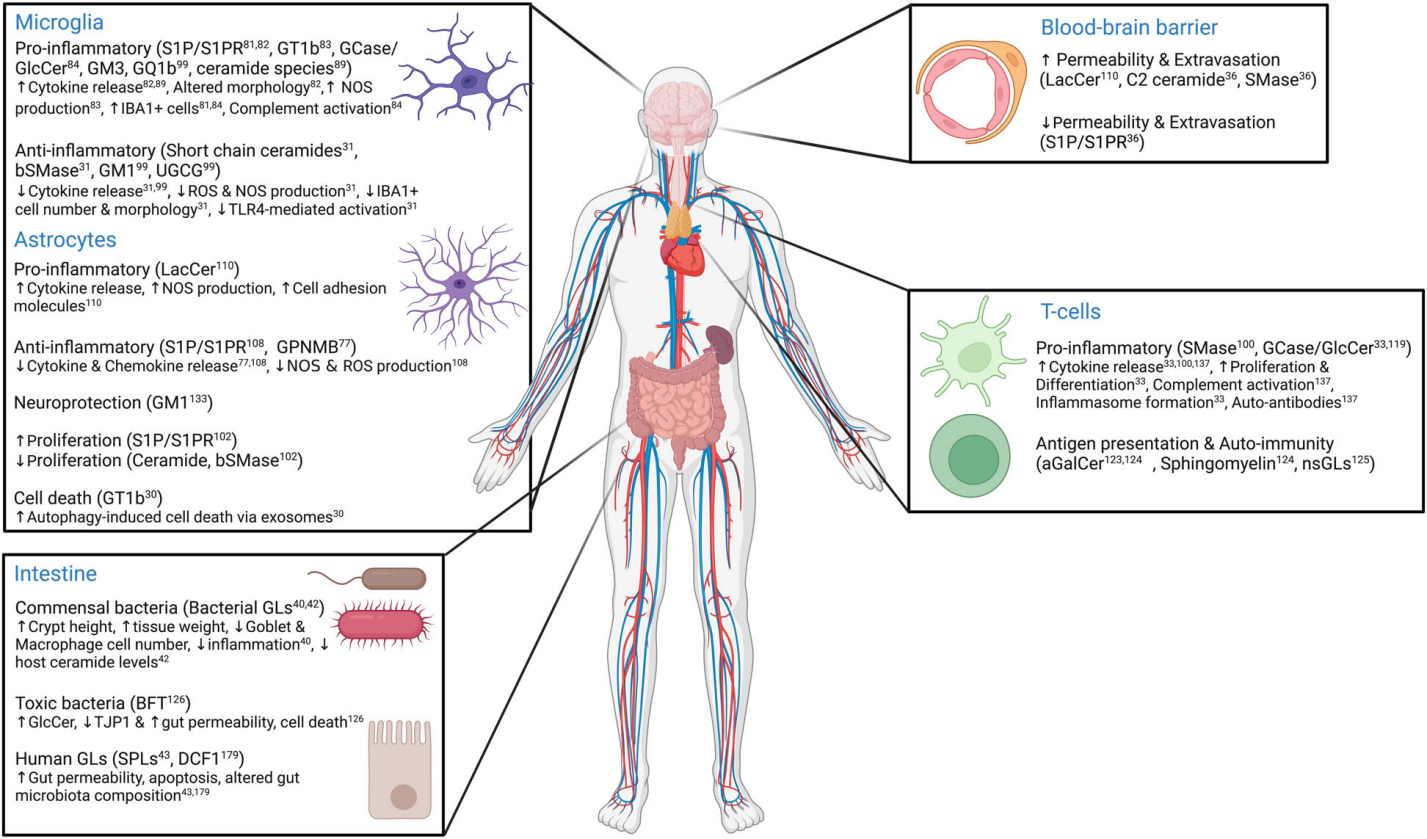
Role of GL in non‐neuronal cell types. GL changes in microglia, astrocytes, BBB, T cells, and in the intestine are associated with altered tissue function. GLs exert both proinflammatory and anti‐inflammatory properties by affecting glial cell activation, cytokine release, proliferation, and cellular migration. GL modulates T‐cell function and antigen presentation. BBB permeability and extravasation are affected by GL changes. Lastly, GLs are key to intestinal function and GL metabolic changes can lead to disruption of the gut barrier and altered microbiota composition. Such pathways could contribute to PD pathogenesis. aGalCer, α‐galactosylceramide; BFT, *Bacteroides fragilis* toxin; bSMase, bacterial sphingomyelinase; DCF1, dendritic cell factor 1; GCase, glucocerebrosidase; GlcCer, glucosylceramide; GPNMB, glycoprotein nonmetastatic melanoma protein B; IBA1, ionized calcium‐binding adaptor molecule 1; LacCer, lactosylceramide; NOS, nitric oxide species; nsGLs, neolacto‐series glycolipids; ROS, reactive oxygen species; S1P, sphingosine 1‐phosphate; S1PR, sphingosine 1‐phosphate receptor; SPLs, sphingolipids; TJP1, tight‐junction protein 1; TLR4, toll‐like receptor 4; UGCG, UDP‐glucose ceramide glucosyltransferase. Created with BioRender.com.

Glycolipids modulate microglial and astrocytic responses during inflammation [29, 30, 31, 32, 33] as well as BBB permeability and immune cell infiltration in the CNS [34, 35]. In the next paragraphs, we summarize the available data regarding the role of GLs in non‐neuronal cells in the CNS and their potential link to PD (Fig. 1, Table 1).

**Table 1:** Summary of glycolipid changes and their effects in different cell types/tissues with relevance to Parkinson's disease. Potential PD‐related consequences are indicated with a question mark. The asterisk depicts GL changes in PD that would have an effect contrary to the one suggested by current experimental evidence. BBB, blood–brain barrier; bSMase, bacterial sphingomyelinase; CSF, cerebrospinal fluid; FTY720, fingolimod; GD, Gaucher's disease; GlcCer, glucosylceramide; iNKT, invariant natural killer T cell; LacCer, lactosylceramide; LPS, lipopolysaccharide; MINCLE, macrophage‐inducible C‐type lectin; PD, Parkinson's disease; S1P, sphingosine‐1‐phosphate; SL, sphingolipid; SN, substantia nigra; SPHK1, sphingosine kinase 1; TLR4, toll‐like receptor 4.

GLs	Change	Effect	Change in PD	PD‐relevant consequences
Microglia				
S1P/SPHK1		**Proinflammatory**	Decrease [58]	Anti‐inflammatory*
	Decrease	↓ IBA1+ cell number [80, 110]		
	Increase	↑ *TNFα*, *IL‐1β*, *IL‐6* [80, 110]		
	Increase	↓ Ramification index, volume, branch number [110]		
	Increase	↓ Migration [82]		
GT1b	Increase	**Proinflammatory**	Decrease [59]	Anti‐inflammatory*
		↑ NOS protein levels [32]		
		iNOS‐induced neurotoxicity [32]		
GlcCer	Increase	**Proinflammatory**	Increase [59, 60]	Proinflammatory
		↑ IBA1+ cell number [83]		
		Complement C1q activation [83]		
		↓ α‐, β‐, γ‐tubulin levels [83]		
Ceramides	Increase	**Proinflammatory**	No difference [61] or decrease [62]	Unknown
		NLRP3 inflammasome formation [88]		
		↑ IL‐1β production [88]		
Short‐chain ceramides (C2–C8)	Increase	**Anti‐inflammatory (in proinflammatory environment – LPS)**	Unknown	Unknown
		↓ TNFα, IL‐1β production [30]		
		↓ NO levels [30]		
		↑ IL‐10 production [30]		
		↓ IBA1+ cell number [30]		
C2 ceramide	Increase	**Anti‐inflammatory**	Unknown	Unknown
		NF‐κB and AP‐1 DNA binding inhibition [30]		
		PKA/CREB activation [30]		
		LPS‐TLR4 interaction inhibition [30]		α‐syn‐TLR4 interaction inhibition?
Long‐chain ceramides (in oligodendrocytes)	Increase	**Proinflammatory**	Decrease in occipital cortex [62]	Anti‐inflammatory*
		↑ IBA+ area [96]		
		Appearance of distinct CD11c+/CD68 microglial population [96]		
GM1, (GD3, GD2, GM2, GD1a, GD1b, and GT1b)	Increase	**Anti‐inflammatory** [98]	Decrease [59, 60, 61]	Proinflammatory
GM3 and GQ1b	Increase	**Proinflammatory** [98]	Increase (GM3) [59]	Proinflammatory
Astrocytes				
S1P	Increase	**Transition to proliferative state** [101]	Decrease [58]	Unknown
	Increase	**Anti‐inflammatory (in proinflammatory environment – LPS)**		Proinflammatory
		↓ *IL‐1*, *IL‐6*, *TNFα*, and *CCL2* [107]		
		↓ Monocyte migration from astrocyte‐conditioned medium [107]		
LacCer	Increase	**Proinflammatory (in proinflammatory environment – TNFα/IFNγ, LPS/IFNγ)**	Increase in CSF and no change in SN [59]	Proinflammatory
		↑ *ICAM‐1*, *VCAM‐1* [110]		
		↑ NF‐κB and STAT‐1 DNA binding [109]		
		↑ iNOS expression [111]		
		↑ TNFα, IL‐1β secretion [111]		
GPNMB	Increase	**Anti‐inflammatory (in proinflammatory environment – TNFα, IL‐1β, and IFNγ)**	Decrease [31]	Proinflammatory
		↓ *IL‐6* [77]		
		↓ NOS and ROS [76]		
		↑ IGF1 and Arginase‐1 [76]		
Ceramide	Increase	**↓ of proliferation** [101]	No difference [61] or decrease [62]	Unknown
Gangliosides (GM1, GT1a, GD1b, GT1b)	Increase	**Astrocyte cell death** [29]	Decrease [59, 60, 61]	Unknown
		Autophagy‐induced cell death [29]		
BBB				
LacCer (astrocytes)	Increase	↑ *ICAM‐1*, *VCAM‐1* [110]	Increase in CSF and no change in SN [59]	Altered BBB function and interaction with peripheral immune cells
C2 ceramide/SMase (endothelial cells)	Increase	Compromised BBB integrity	Unknown	Compromised BBB integrity
		↓ Transendothelial electric resistance [35]		
		↑ Monocyte migration [35]		
S1P (FTY720)	Increase	Rescued BBB function in MS models [112, 113, 114]	Decrease [58]	Impaired BBB function
T‐cells				
GlcCer	Increase	**MINCLE‐dependent proinflammatory transition**	Increase [59, 60]	Proinflammatory
		ASC‐NLRP3 inflammasome activation [38]		
		IL‐1β secretion [38]		
		Th17 cell proliferation [38]		
GlcCer	Increase	**C5a‐induced T‐cell activation**	Decrease in C5a [115, 116, 117, 118]	Anti‐inflammatory*
		↑ IL‐1β, IL‐6, TNFα, and IL‐17 secretion [118]		
		↑ IFNγ production [118]		
		↑ CD40L [118]		
		Production of IgG GlcCer autoantibodies [118]		Increased IgG autoantibodies in GD patients' serum [118]
iNKT				
Ceramides and cholesterol	Increase under ER stress	**Lipid‐induced autoimmune responses** [122]	No difference or decrease [61, 62]	Altered autoimmune responses against lipids*
		↓ α‐glucosidase, α‐galactosidase, and acid ceramidase gene transcription [122]		
Sphingomyelin	Increase	**Anti‐inflammatory (antigen presentation regulation)** [123]	Increase in SN, decrease in CSF [61, 115]	Altered autoimmune responses against lipids
		↓ aGalCer‐mediated IL‐2 release [123]		
		Displacement of CD1d‐bound molecules [123]		
nsGLs	Increase	Anti‐inflammatory (antigen‐antibody interaction) [124]	Unknown	Unknown
Intestine				
Bacterial SLs	Decrease	↑ Crypt height, intestinal tissue weight, goblet, and macrophage cell numbers [39]	Reduced *Bacteroidetes* [119, 120]	Impaired gut function
		Altered host cecal ceramide composition and levels [39]		
GlcCer	Decrease	**Gut barrier disruption under BFT** [125]	Unknown	Unknown
Host SLs	Decrease	Diarrhea, rectal bleeding, gut barrier permeability, ↓ MUCIN2 [42]	Increase in Cer(d14:1(4E)/22:0) [121]	Unknown
		Caspase‐3 cleavage, apoptosis [42]	Decrease in Cer(d18:0/14:0) [121]	
		↑ Proliferation [42]		
		↑ TNFα and IL‐6 levels [42]		

## Glycolipids in microglia

Glycolipids play key roles in microglia, including (a) the synthesis of inflammatory cytokines and eicosanoids, (b) antigen presentation, and (c) phagocytic clearance [78]. In PD and in aging, microglia accumulate neutral lipids [75, 79], and individual GLs may exert both pro‐ and anti‐inflammatory roles.

Several studies support the proinflammatory role of S1P/S1P receptor (S1PR) signaling in microglia. The inhibition of S1P/S1PR with the antagonist JTE013 in a mouse ischemic brain injury model leads to a reduced number of IBA1+ microglia, decreased microglia proliferation, and the downregulation of *Tnfα*, *Il‐1β*, and *Il‐6* gene expression [80]. In contrast, the activation of the S1P/S1PR axis by genetically ablating *Sgpl1*, which catabolizes S1P, in mouse neurons is associated with microglial activation [81]. Specifically, the microglial ramification index, volume, and number of branches are reduced in the cerebellum, cortex, and hippocampus of neuronal *Sgpl1* ^*−*/*−*^ mice [81], suggesting their transition to a proinflammatory phenotype. S1P treatment also induces microglial migration in BV2 cells [82]. Primary microglia from *Sgpl1* ^*−/−*^ mice treated with rapamycin show decreased IL‐6 secretion, suggesting the role of autophagy in the S1P‐induced microglial response [81]. One of the most abundant CNS GLs, GT1b, also plays a proinflammatory role. The injection of GT1b in the rat SN leads to microglial activation with a concurrent increase in inducible nitric oxide synthase (iNOS) protein levels, specifically in microglia [32]. This change is followed by oxide‐induced neurotoxicity, as l‐NAME, a NOS inhibitor, partially rescues SN DA neuron death. However, because GT1b is reduced in the PD SN [59], further studies are necessary to understand whether GT1b is proinflammatory in the PD brain. Experimental models of familial PD also support a link between GLs and immune pathways. A chronic 28‐day inhibition of GCase in mice by CBE induces widespread microglial activation, including SN and striatum, as measured by the number of IBA1+ microglial cells and complement C1q activation [83]. Microglial activation persists even after 7 days in the absence of CBE, suggesting a prolonged effect. However, the GL burden and GCase activity have not been measured. CBE treatment also induces the formation of α‐syn aggregates [83]. Interestingly, the cytoskeletal proteins α‐, β‐, and γ‐tubulin are reduced in whole‐brain lysates. These findings can be linked to the loss of cortical neurons in this model. Because tubulins are also involved in microglial response and migration [84, 85], investigating whether microglia have reduced migratory ability would be interesting. Finally, damaged cells release β‐GlcCer, which is a ligand of macrophage‐inducible C‐type lectin (MINCLE) [36], a receptor involved in pathogen‐ and damage‐associated molecular pattern recognition [86]. Interestingly, a very recent report showed that β‐GlcCer accumulation activates microglia to induce the phagocytosis of living neurons in a GD model [87].

Recent findings have revealed that ceramide species are positive modulators of NLRP3 inflammasome assembly and IL‐1β production [88]. The induction of ceramide signaling by C2 ceramide and fingolimod (FTY720), an S1PR agonist, led to ASC speck formation in mouse primary cultures [88]. Both C2 ceramide and sodium palmitate, a serine palmitoyl transferase (SPT) activator (SPT is an enzyme involved in *de novo* ceramide synthesis [89]), trigger IL‐1β release [88]. The effect is modulated by the ASC pathway because *Asc* ^*−/−*^ microglia do not have increased IL‐1β release following C2 ceramide stimulation. These effects are not due to ceramide transport because transport inhibition does not rescue IL‐1β secretion and caspase‐1 cleavage [88]. With regard to genetic models, 9‐week‐old *Pink1* ^*−/−*^ mice display pronounced glial activation accompanied by increased levels of several ceramide species (C16, C18, C18:1, C20, C24, and C24:1 ceramides, C18 glucosylceramide, S10, sphingosine and C18, C24:1 lactosyl‐ceramides) in the olfactory bulb. Interestingly, *Pink1* ^*−/−*^ mice reach a plateau of total ceramide levels in the brain from 9 weeks of age, while wild‐type (WT) mouse ceramide levels increase with age and are equal to those of 21‐week‐old *Pink1* ^*−/−*^ mice [90]. Because PINK1 is a mitochondrial kinase with a direct role in protein quality control and immunity [91], dissecting whether and how GL alterations trigger microglial and astrocytic responses in *Pink1* ^*−/−*^ mice would be important [90]. However, ceramides could be associated with neuroinflammation observed in aged *Pink1* ^*−/−*^ mice.

Ceramide and other GLs also play anti‐inflammatory roles in microglia. BV2 and rat primary lipopolysaccharide (LPS)‐stimulated microglia treated with short‐chain ceramides (C2, C6, C8, or C8‐ceramide‐1‐phosphate) show reduced TNFα and IL‐1β production, reduced NO levels, and increased lL‐10 secretion compared with LPS‐only treated microglia [30]. Notably, C2 ceramide plays an anti‐inflammatory role in this experimental setting [30]. However, this finding is in contrast with that of another study showing a proinflammatory role for C2 ceramide in mouse primary microglia [88]. These discrepancies could be attributed to the model system, namely BV2 and rat primary microglia [30] and mouse primary microglia [88]. Furthermore, while both studies used C2 ceramide, the ceramide pathway is manipulated differently, namely using C2 directly or bacterial sphingomyelinase (bSMase) in the former [30] and sodium palmitate or FTY720 or the ceramide‐to‐sphingomyelin inhibitor HPA‐12 in the latter [88]. Therefore, it would be crucial to determine how different approaches to modulating the ceramide pathway influence microglial inflammatory responses as well as the role of the ceramide pathway in human microglia.

Concerning the anti‐inflammatory role of C2 ceramide, the number of IBA1+ microglia is reduced when mice are pretreated with C2 ceramide prior to systemic injection of LPS compared with LPS‐injected mice [30]. Interestingly, microglial total ceramide levels only increase upon treatment with C2‐ceramide but not in C8‐C1P‐ or bSMase‐treated cells [30]. Treatment with bSMase leads to increased levels of the long‐chain ceramides C16:0 and C24:1 but not C2 ceramide [30]. These findings suggest that C2 ceramide is not converted into long‐chain ceramides. Thus, C2 ceramide could have anti‐inflammatory effects as it enters microglia. However, the study shows evidence of bSMase also being anti‐inflammatory because bSMase treatment results in reduced TNFα secretion and reactive oxygen species (ROS) and NO production in LPS‐stimulated BV2 microglia [30].

With regard to the involved molecular pathways, C2 ceramide inhibits the DNA binding and activity of the proinflammatory inducers NF‐κB and AP‐1 [30]. In line with these data, the PKA/CREB pathway is activated in C2 ceramide LPS‐stimulated microglia compared with LPS‐only stimulated microglia, as measured by CREB DNA binding, activity, and nuclear translocation [30]. This pathway is involved in the resolution of inflammation and in the upregulation of the antioxidant modulator HO‐1 in C2 ceramide LPS‐stimulated microglia. Furthermore, C2 ceramide can block the interaction of LPS with Toll‐like receptor 4 (TLR4) [30]. α‐syn can interact with TLR4 and trigger microglial and astroglial activation [92] and microglial‐mediated α‐syn clearance [93]. Hence, a plausible hypothesis would be that C2 ceramide could inhibit the interaction of α‐syn with TLR4, leading to reduced α‐syn uptake by microglia. Thus, short‐chain ceramides seem to play anti‐inflammatory roles in microglia [94].

Mice deficient for *Cers2*, a ceramide synthase [95], specifically in oligodendrocytes, display reduced C22‐24 in myelin followed by an increase in the long‐chain C16‐18 ceramides [96]. Microglia are activated, as evidenced by an increased IBA1+ area in the corpus callosum, cerebellum, and striatum as well as the appearance of a distinct CD11c+/CD68+ microglial population in *Cers2* ^*−/−*^ versus WT mice [96]. Further understanding how different chain‐size ceramides specifically modulate immune cells could help identify therapeutic strategies for PD.

GM1 has been shown to be neuroprotective by attenuating α‐syn aggregation and DA neuronal loss in rats overexpressing adeno‐associated virus‐(AAV) A53T‐α‐syn in the SN [97]. Interestingly, in another study, GM1 treatment also attenuated the activation of BV2 microglia and mouse primary microglia [98] as well as microglial responses in AAV‐A53T rats [33, 98]. Both the sialic group and ceramide tail contribute to the anti‐inflammatory properties of GM1 [98]. GD3, GD2, GM2, GD1a, GD1b, and GT1b gangliosides also display anti‐inflammatory functions [98]. In contrast, GM3 and GQ1b are proinflammatory, and GM1 can antagonize GD3 action [98]. Based on the different structures of GM3 and GQ1b, future studies should address the structural components and downstream cellular pathways responsible for the proinflammatory responses compared with the other gangliosides tested. Furthermore, an interesting hypothesis could be that GLs play a concentration‐ and subcellular localization‐dependent role in immune cells. Relevant to PD, where gangliosides are reduced [59, 60, 61], blocking UDP‐glucose ceramide glucosyltransferase (UGCG), an enzyme involved in the synthesis of ganglioside precursors, causes increased IKK and p38‐MAPK phosphorylation upon LPS stimulation in BV2 microglia as well as increased *Tnf* and *Il1‐β* expression in BV2 microglia and mouse primary microglia [98]. Of note, UGCG inhibition leads to reduced GM1 and GD1a and an increase in GT1b levels without changes in GD1b levels [98]. These results suggest the involvement of TLR4 and the NF‐κB pathway in the inflammatory responses regulated by gangliosides. The NF‐κB pathway is also regulated by ceramides, suggesting that it could potentially be targeted to reduce neuroinflammation in PD [30, 99]. With regard to human disease, PD brains show reduced S1P levels and a decline in the expression and activity of sphingosine kinase 1 (SPHK1) [58]. Interestingly, conditional *Sphk1* deficiency in neurons contributes to defects in phagocytosis and resolution of inflammation in Alzheimer's disease (AD) models [100].

## Glycolipids in astrocytes

The dual role of GLs in astrocytes is similar to that described in microglia. S1P can act as a signaling molecule to trigger primary rat astrocyte proliferation via ERK phosphorylation [101]. Basic fibroblast growth factor (bFGF) treatment in rat astrocytes leads to enhanced S1P secretion to transition astrocytes from a quiescent to a proliferating state [101]. Basic FGF is protective by reducing *Gfap* expression and the secretion of TNFα and IL‐6 cytokines [102]. Studying whether and how S1P cooperates with bFGF as a potential anti‐inflammatory molecule in astrocytes could help identify novel therapeutic targets. On the contrary, ceramide reduces proliferation and ERK phosphorylation in S1P‐treated astrocytes [101]. The same effect is observed in astrocytes treated with bSMase [101]. Because ERK plays a role in both astrocyte development and activation [103, 104, 105, 106], further studies are needed to elucidate whether S1P and ceramide are pro‐ or anti‐inflammatory in astrocytes in the context of PD.

FTY720, an S1PR activator, can signal murine LPS‐treated astrocytes to reduce the expression of proinflammatory genes, including *Il‐1*, *Il‐6*, *Tnfα*, and *Ccl2* [107]. CCL2 is a chemokine involved in monocyte/macrophage recruitment [108]. Indeed, conditioned medium from LPS‐ and FTY720‐treated mouse astrocytes reduces the migratory potential of monocytes compared with conditioned medium from LPS‐treated astrocytes [107]. Another GL, LacCer, also seems to play a role in modulating astrocyte morphology and function. LacCer treatment increases the expression of the cell adhesion molecules *Icam‐1* and *Vcam‐1* in TNFα‐ and IFNγ‐stimulated astrocytes [109]. Blocking the LacCer synthesis enzyme GALT‐1 with d‐Threo‐1‐phenyl‐2‐decaanoylamino‐3‐morpholino‐1‐propanol (PDOP) or *N*‐butyldeoxynojirimycin (NBDNJ) prevents this upregulation [109]. LacCer‐induced responses are likely mediated by NF‐κB and STAT‐1 binding to the promoters of *Icam‐1* and *Vcam‐1*, as shown using the luciferase reporter system [109]. The same GL induces iNOS expression and TNFα and IL‐1β secretion via the NF‐κB pathway in primary mouse astrocytes [111]. Blocking LacCer synthesis with PDMP can reduce astrogliosis in spinal cord injury mouse models [111]. Therefore, the proinflammatory effects of LacCer may be linked to the NF‐κB pathway.

GPNMB, an endogenous glycoprotein that has been recently linked to inflammation [76, 126] and the LSDs GD and NPC, is upregulated in PD [31, 75, 76, 77, 127, 128, 129]. Despite this evidence, studies thus far have not addressed whether and how GLs may directly lead to increased *GPNMB* expression. GPNMB protein levels increase in astrocytes and microglia in the SN of CBE‐treated *Thy1*‐α‐syn mice [31] and in astrocytes in the striatum of 1‐methyl‐4‐phenyl‐1,2,3,6‐tetrahydropyridine (MPTP)‐treated mice [76], suggesting that it may be involved in inflammatory pathways in PD. GPNMB is normally cleaved and leads to the formation of an extracellular soluble protein fragment. Mouse astrocytes treated with the cytokines TNFα, IL‐1β, and IFNγ and a recombinant extracellular fragment of GPNMB have attenuated *Il‐6* expression, nitrogen species, and ROS production compared with astrocytes treated only with TNFα, IL‐1β, and IFNγ [76]. Furthermore, the extracellular fragment of GPNMB has an anti‐inflammatory role, as it reduces *Il‐6* expression below baseline levels and increases IGF1 and Arginase‐1 anti‐inflammatory proteins in cultured mouse astrocytes compared with untreated astrocytes [76]. In line with the experimental model, GPNMB protein levels are increased in the SN of PD patients [31, 76].

Interestingly, changes in ceramides and GLs can also trigger astrocyte cell death [29, 130]. A mixture of the brain gangliosides GM1, GT1a, GD1b, and GT1b can lead to mouse astrocyte cell death, with GT1b playing a key role [29]. Ganglioside treatment caused the conversion of LC3‐I to LC3‐II and increased the fluorescence of monodansylcadaverine (MDC) [29], a marker of autophagic vacuoles. The inhibition of autophagy with 3‐methyladenine or *Atg6* or *Atg7* knockdown attenuates ganglioside‐induced astrocyte death [29]. Inhibiting mTOR with rapamycin exacerbates astrocyte cell death upon ganglioside treatment, whereas ERK inhibition with PD98059 rescues the effect [29]. Additionally, lipid raft disruption with MβCD inhibits cell death and partially rescues MDC fluorescence [29]. However, the type of cell death pathway was not analyzed. Evaluating whether such astrocyte death mechanisms are present in experimental models of neurodegeneration and humans would be important. With relevance to neurodegeneration, amyloid‐β triggers ceramide and PAT‐4 production and apoptosis in astrocytes both *in vitro* and *in vivo* using the 5xFAD model [130]. Supporting ceramide's involvement in apoptosis and the role of GLs as signaling molecules in neurodegeneration, neutral SMase^*−/−*^ mouse primary astrocytes are protected from cell death [130]. Future investigations should address whether these GL/ganglioside pathways are involved in inflammation and apoptosis in human PD models and PD brains.

Astrocyte play key roles in supporting neuronal metabolism and they also exert neuroprotective functions [131]. Treatment of mouse primary astrocytes with GM1 leads to glucose uptake and downstream lactate secretion without altering mitochondrial function and redox status [132]. Additionally, GM1 induces the expression of the glucose metabolism‐related genes *Ptg*, *Hexokinase*, *Pdh*, and *Taldo* [132]. Moreover, GM1‐treated astrocytes [132] upregulate the neuroprotective genes *Arc*, *Egr4*, and *Nr4a3* [132] and display a neuroprotective role in cocultures upon glutamate‐induced neuronal death [132]. Interestingly, GM1 enhances neuronal mitochondrial activity and exerts neuroprotection when neurons are cocultured with astrocytes [132]. These data highlight the key role of GM1 in astrocyte‐related functions. As GM1 levels are reduced in PD [59, 60], astrocytes may not be able to support the high metabolic demand of SNpc DA neurons.

## Glycolipids in the regulation of the blood–brain barrier

The BBB functions to prevent blood molecules and cells from freely entering the brain and consists of endothelial cells that interact with astrocytes and pericytes [133]. BBB permeability is altered in neurodegenerative diseases, including PD [134, 135]. Mice with mutations in genes involved in LSDs (*Gba1*, beta‐hexosaminidase, and beta‐galactosidase‐1) have compromised permeability or extravasation [136, 137]. Astrocytes also play a key role in BBB function. The cell adhesion molecules *Icam‐1* and *Vcam‐1* are upregulated in LacCer‐treated astrocytes prestimulated with TNFα and IFNγ (mechanisms described in the previous section) [109]. These molecules are important for BBB function, astrocytic binding, and interaction with peripheral immune cells [138, 139]. The effect of GLs has also been studied in brain endothelial cells. Human CMEC/D3 cells treated with C2‐ceramide or SMase, which increases intracellular ceramide levels, show compromised BBB integrity, as measured by transendothelial electric resistance [35]. BBB integrity loss due to C2‐ceramide or SMase also leads to increased human blood monocyte passage through the hCMEC/D3 monolayer [35]. Additionally, conditioned medium from TNFα‐treated primary human astrocytes also leads to the increased migration of monocytes through hCMEC/D3 compared with conditioned medium from untreated astrocytes [35]. The cotreatment of astrocytes with TNFα and FTY720 rescues the increased migration observed by treatment with TNFα only [35]. FTY720 is a drug used for the treatment of the autoimmune disease multiple sclerosis (MS) [112]. The BBB is disrupted in both MS patients and experimental models of MS, termed experimental autoimmune encephalomyelitis (EAE) [140]. Indeed, FTY720 prevents the commitment of T cells to proinflammatory Th17 cells and CNS infiltration in EAE rat models [112, 113, 114]. Thus, controlling ceramide levels is key to BBB function.

## Glycolipids beyond the CNS

Glycolipids display diverse biological functions that are key to the homeostasis of several tissues and organs. Here, we will discuss the role of GLs in the peripheral immune system and in the gut, which may be relevant to PD.

### Glycolipids in T‐cell function and autoimmunity

Ceramide levels and neutral SMase activity increase during aging and mediate the cellular senescence of multiple cell types, including macrophages and T cells [37, 141]. In T cells, TNFα binding to the TNF receptor causes phosphatidylcholine cleavage to diacylglycerol, which directly activates acidic SMase (aSMase). Ceramide produced by aSMase triggers NF‐κB DNA binding and proinflammatory responses in macrophages [99]. Increased ceramides in macrophages trigger the COX‐2‐PGE2 pathway via NF‐κB, which inhibits T‐cell function [142, 143, 144]. The ceramide‐induced upregulation of COX2 is further exacerbated by LPS stimulation in mice [37], suggesting that prestimulated T cells are more responsive to ceramide alterations [37]. Because COX‐2 is also linked to neuroinflammation [145], future studies should address how ceramide affects COX‐2 in the CNS. In addition to ceramide, GLs also modulate T‐cell function [38, 118, 123, 124]. β‐GlcCer is a natural ligand for MINCLE [36].

In an EAE model, dying cells released β‐GlcCer, which binds MINCLE on Th17 cells, leading to ASC‐NLRP3 inflammasome activation, caspase‐8‐dependent IL‐1β production, and consequent Th17 cell proliferation via an autocrine regulatory loop [38]. Inhibiting β‐GlcCer synthesis with AMP‐DNM protects mice from EAE progression and dampens immune cell infiltration into the CNS [38]. While Th17 cells are relevant to autoimmune diseases, their role in CNS inflammation and effect on BBB function can make their responses to β‐GlcCer relevant to PD. Interestingly, GD Type I patients have increased T‐suppressor and cytotoxic T cells (CD8+HLA‐DR+), activated T‐helper cells (CD4+HLA‐DR+), and activated T‐lymphocytes (CD3+HLA‐DR+) compared with children without a *GBA1* mutation [146]. The infiltration of peripheral immune cells, termed Gaucher cells, is present in the brain perivascular space of GD patients [147]. Although Gaucher cells are not detected in PD, T cells may infiltrate the CNS [148, 149, 150]. This behavior is in line with the potential role of GL in the BBB dysfunction observed in PD. Thus, GL imbalance could cause the recruitment of peripheral immune cells in the CNS, exacerbating the neuroinflammatory environment.

Complement‐5a (C5a) and its receptor C5aR1 are increased in mouse‐derived macrophages and dendritic cells (DCs) from *Gba1* ^*9V/−*^ mice [118]. C5a leads to elevated CD40 protein levels in CD4+ T cells in *Gba1* ^*9V/−*^ mice compared with WT mice. C5a treatment of DCs cocultured with CD4+ cells carrying *Gba1* ^*9V/−*^ resulted in higher IL‐1β, TNF, IL‐6, and IL‐17 secretion compared with WT cells [118]. Long‐term CBE injection leads to the production of IFNγ and IL‐17, increased CD40L expression, β‐GlcCer accumulation in macrophages, and premature death in WT mice. *C5ar1* ^*−/−*^ protects from CBE‐induced death, even 60 days after injection [118]. In *Gba1* ^*9V/−*^ mice treated with a C5aR1 antagonist (C5aRa), macrophages from *Gba1* ^*9V/−*^/*C5ar1* ^*−/−*^ mice show reduced levels of glucosylceramide species C16:0, C22:0, C24:0, and C24:1 compared with *Gba1* ^*9V/−*^ macrophages [118]. β‐GlcCer accumulation leads to the production of autoimmune IgG2a/c and IgG2b autoantibodies that trigger downstream C5a responses. In line with these data, IgG1 and IgG3 levels as well as IgG2 and C5a are increased in the serum of GD patients [118]. However, PD patients show a decrease in C5a levels in the CSF, while serum C5a levels are comparable between PD patients and healthy controls [117]. Due to β‐GlcCer accumulation and the inflammatory environment, C5a would be expected to be increased. Unexpectedly, the CSF C5a concentration and CSF β‐GlcCer d18:1/C23:0 levels correlate in PD patients [117]. Moreover, the CSF levels of β‐GlcCer did not change in PD patients in this study [117]. However, the results relative to β‐GlcCer levels in PD CSF are inconclusive. Another study has shown that total β‐GlcCer levels increase, and total sphingomyelin levels decrease in the CSF of *GBA1*‐PD patients compared with controls and idiopathic PD patients [115]. Finally, a study reports increased β‐GlcCer species in the CSF of both *GBA1*‐PD and idiopathic PD patients compared with controls [116]. Thus, consensus regarding β‐GlcCer alterations in PD CSF is lacking, highlighting the large heterogeneity of PD cases. Changes in the levels of GLs and GL‐related enzymes in the CSF or blood of PD patients compared with controls are described in detail in the review of Belarbi *et al*. [34]. Thus, evidence of GL imbalance in GD patients [146] and the altered GL profile of PD patients [59, 151] highlight that peripheral immune cells can impact inflammation and cell activation in the CNS.

Glycolipids are involved in antigen presentation and autoimmunity pathways [152, 153]. Interestingly, IgG antibodies are produced against β‐GlcCer in *GBA1* ^9v/*−*^‐ and CBE‐mouse models, and these antibodies have also been found in the serum of GD patients [118], suggesting that autoimmune responses against GLs could be relevant to PD. Natural killer T (NKT) cells recognize processed fragments of complex GLs presented in the context of the nonpolymorphic MHC class I‐like molecule CD1d [154, 155]. The presentation of GLs by CD1d was revealed almost 20 years ago when the structures of mouse and human CD1d molecules were determined in complex with α‐galactosylceramide [156, 157].

CD1‐restricted T cells play key roles in infection, cancer, and autoimmunity. Defects in lipid antigen presentation by CD1d have been described in mouse models of LSDs, including Sandhoff, NPC, GM1 gangliosidosis, and Fabry disease [158, 159, 160, 161, 162]. Such changes are accompanied by a defect in the percentage of both thymic and peripheral invariant NKT (iNKT) cells [159, 160, 161, 162, 163, 164]. Although Fabry patients show a reduction in the CD4+ and an increase in the double‐negative iNKT populations, no differences are detected in the frequency of total iNKT cells in NPC disease, Fabry disease, and GD [165, 166, 167]. With regard to GD, β‐GlcCer 22:0 (βGL1‐22) and glucosylsphingosine are recognized by a distinct subset of CD1d‐restricted human and murine type II NKT cells, which could lead to the chronic B‐cell activation and gammopathy observed in these patients [167]. However, these pathways may also play a role in the pathogenesis of PD to different extents. Several cellular mechanisms may affect lipid antigen presentation by human CD1 molecules in LSDs, including abnormal substrate accumulation in the lysosomes/late endosomes as well as defects in endolysosomal trafficking and autophagy [168].

Furthermore, ER stress, which is observed in PD patients and human iPSC‐derived DA neurons with *GBA1* mutations [16, 17, 169, 170], could also play a role. Monocyte‐derived dendritic cells (MoDCs) treated with thapsigargin, which induces ER stress, trigger the release of IFNγ and IL‐12p40 from cocultured iNKT cells [122]. ER stress signals via the PERK pathway to induce CD1d‐mediated antigen MoDC presentation to iNKTs [122]. The lipid species that elicit immune responses are cholesterol and ceramide species. Furthermore, ER stress reduces the transcription of α‐glucosidase, α‐galactosidase, and acid ceramidase, all of which participate in GL catabolism [122]. Interestingly, ER stress also affects actin‐mediated CD1d organization on the cell surface [122], suggesting that multiple pathways are affected during ER stress, leading to enhanced inflammation. These data suggest a link between ER stress, GL metabolism, and autoimmunity, which could be relevant to PD.

In addition to being immunogenic, GLs directly modulate the process of antigen presentation [123, 124]. aSMase, which degrades sphingomyelin, plays a key role in iNKT cell development. Indeed, aSMase‐deficient mice have a reduced number of iNKT cells at developmental stages 1‐3 in the thymus and total iNKT cell number in the thymus, spleen, and liver [123]. As expected, sphingomyelin species, but not ceramide species, accumulate in aSMase knockout mice. Interestingly, CD1d‐bound α‐galactosylceramide (aGalCer)‐mediated IL‐2 release from iNKTs is attenuated by increasing concentrations of different sphingomyelin species (C24:1, C16:0, C18:0, and C24:0) [123]. Indeed, these sphingomyelin species are increased in lysosomes from aSMase‐deficient mice [123]. These results suggest that sphingomyelin competes with immunogenic self‐lipids to reduce the inflammatory response of iNKTs. This study also suggests that sphingomyelin displaces CD1d‐bound molecules, although the ability to do so depends on the binding capacity of the molecule to CD1d [123]. Consequently, identifying the additional proinflammatory GLs that are exposed by CD1d and whether sphingomyelin has a similar ability to displace them would be interesting. Likewise, neolacto‐series GLs (nsGLs) suppress immune recognition and responses. The knockout of the protease SPPL3 in HAP1 cells caused enhanced HLA‐1‐mediated CD8+ cytotoxic T‐cell activation [124]. Downstream genome‐wide haploid screening combined with antibody binding capacity to HLA‐1 reveals the involvement of the GL pathway. UGCG, B4GALT5, and B3GNT5, which are enzymes that catalyze the formation of GLs in the Golgi membrane, were identified in the screen [124]. SPPL3 directly interacts with and inactivates B3GNT5, which is the first enzyme in the synthesis of lacto‐ and nsGLs [124]. The mechanism of decreased HLA‐1 receptor accessibility occurs via the sialic group of nsGLs [124]. While this study focused on cancer treatment, the nsGL pathway could be a potential target for immunosuppression, similar to sphingomyelin. However, the inflammatory responses leading to increased GL levels in LSDs, and possibly PD, are due to the accumulation of multiple GLs. Because both sphingomyelin and ceramide levels increase in PD, investigating how ceramides compete with the anti‐inflammatory function of sphingomyelin would be relevant. The levels of different gangliosides are also altered (i.e., GM1a, GD1a, GD1b, and GT1b) [59, 60, 66], adding an additional degree of complexity to the autoimmunity hypothesis. The applicability of these mechanisms of antigen presentation and competition to PD and their ability to be modulated are important areas of study.

### Glycolipids in the gut and gut microbiota

The gut microbiome is an important source of GLs in the body. Specifically, the *Bacteroidetes* phylum, which is abundant in the human intestine, can synthesize GLs [40]. Caco‐2 cells cocultured with *Bacteroidetes* uptake bacterial‐synthesized GLs and induce changes in host GL metabolic gene expression, including *SPHK1*, the sphingomyelinases *SMPD2* and *SMPDL3B*, and the ceramide synthase *CERS1* [41]. In mice, bacterial‐derived sphingolipids modulate the levels of bioactive lipids in the liver [41]. The fate of bacterial GLs and their role in humans has been described in detail in the review by Heaver *et al*. [40]. The following section describes the roles of bacterial‐ and host‐produced GLs in gut function. It should be pointed out that these effects could be partially modulated by enteric neuron responses, a cell type that is densely populating the intestine [171].

The gut microbiome is significantly altered in PD patients compared with healthy controls [172]. Ceramides Cer(d14:1(4E)/22:0(2OH)) and sphingosine as well as enzymes involved in glucosylglycerate biosynthesis are elevated in stool samples of PD patients compared with controls, while ceramide Cer(d18:0/14:0) is decreased [121]. Constipation is also associated with changes in various lipids, including sphingolipids [121]. While the exact roles of distinct GLs need to be explored, studies highlight their relevance in host‐microbiota homeostasis and in IBD. As observed in PD, IBD patients display altered sphingolipid profiles in stool samples [39]. Additionally, IBD patients have reduced intestinal expression levels of *SPTLC2*, the second subunit of the SPT enzyme that is the rate‐limiting enzyme in sphingolipid synthesis [89]. Bacterial sphingolipids negatively correlate with host sphingolipid production and inflammation in IBD [39]. Germ‐free (GF) mice colonized with sphingolipid‐deficient *Bacteroides* (*SPT* deletion) have increased crypt height and intestinal tissue weight and elevated goblet and macrophage cell numbers [39]. The host cecal ceramide levels and composition are also altered. Therefore, bacterial sphingolipids are key to gut function, and the lack of sphingolipid‐producing commensal bacteria can lead to abnormal inflammatory responses. While *Bacteroidetes* are commensal bacteria, *Bacteroides fragilis* is toxic to the host because it produces the *B. fragilis* toxin (BFT) [173]. BFT increases the levels of β‐GlcCer in mice and mouse‐derived colonic organoids and upregulates β‐GlcCer synthase (*Gcs*) gene expression [125]. Inhibiting GCS with ibiglustat upon BFT treatment causes organoid collapse via tight‐junction protein 1 (TJP1) mislocalization, reduced TJP1 levels, and cell death (shown using cleaved caspase 3) [125]. Increasing the levels of β‐GlcCer using CBE rescues this phenotype [125]. These data suggest that β‐GlcCer acts as an important molecule for BFT‐induced host responses by reducing cell death and gut permeability. In another study, the role of host‐produced intestinal sphingolipids in normal gut structure and function was addressed [42]. Intestinal conditional *Sptlc2* ^*−/−*^ leads to a series of intestinal complications, namely, diarrhea and rectal bleeding, gut barrier permeability, caspase‐3 cleavage, apoptosis, and increased proliferation [42]. The levels of MUCIN2 are decreased, and LPS is found in the peripheral blood along with increased levels of TNFα and IL‐6 in *Sptlc2* ^*−/−*^ versus WT mice, further indicating a dysfunctional gut barrier and peripheral inflammation [42]. Knockout mice die after 7‐10 days, highlighting the importance of gut GL metabolism in host health [42]. Treating *Sptlc2* ^*−/−*^ mice with antibiotics and dexamethasone rescues mouse death, although the effect of either antibiotics or dexamethasone has not been investigated. With relevance to PD, *Dcf1* ^*−/−*^, a gene involved in nervous system development [174] and α‐syn degradation [175], alters the gut microbiome composition of mice and leads to decreased *Prevotellaceae*, similar to that observed in PD [176]. This family is associated with metabolic disorders of GLs [177]. In summary, both host‐ and *Bacteroides*‐derived GLs are important for normal gut function, and alterations caused by infections or changes in microbiota composition can have a significant effect on gut permeability and inflammatory responses, which could be relevant to PD pathogenesis.

## Future perspectives and therapeutic implications of glycolipids in PD

Glycolipid imbalance affects multiple cell types in the human body and changes in GLs naturally occur during aging. PD patients show changes in GL levels both in the brain (neurons, astrocytes, and microglia) and in the periphery (peripheral immune system, intestinal cells, and gut microbiota). Several mechanisms involved in cellular responses caused by GL imbalance have been studied, including the proinflammatory transition of immune cells. However, their actual role in PD pathogenesis remains to be elucidated. Furthermore, recent evidence shows that GLs play a key role in intestinal function and gut barrier permeability. Future studies will address whether and how these mechanisms are involved in PD. Dissecting the role of GLs in PD would help identify novel biomarkers and therapeutic targets. Substrate reduction therapies have already been tested in GD and PD patients [178, 179]. Other sphingolipid pathways could also be targeted, such as S1P. Reduced levels of S1P levels as well as decreases in *SPHK1* expression and SPHK1 activity have been described in PD [58]. S1P dampens apoptosis in MPP‐treated human neurons [180]. Similarly, FTY720 (an agonist for S1PR_1_), reduces DA neuron loss and attenuates behavioral deficits in chemical‐induced PD mouse models [181, 182, 183]. Interestingly, the S1P signaling axis also targets mitochondrial function, which is impaired in PD [184]. S1P treatment enhances the expression of the mitochondrial‐related genes *PGC‐1α* and *NRF‐1*, which are reduced in non‐S1P‐treated MPTP‐induced PD mice [185]. S1P signaling pathway can additionally regulate neuroinflammation via COX2 [100, 145]. COX2 is involved in microglial phagocytosis and inflammatory resolution in AD and COX2 acetylation (its activated form) is mediated via SPHK1 [100]. As *SPHK1* expression and SPHK1 activity levels are reduced in both AD and PD [58], SPHK1 could be a potential therapeutic target for neurodegenerative disorders. Besides sphingolipids, gangliosides may have disease‐modifying effects in PD [186, 187]. Clinical trials have been conducted to assess the therapeutic role of GM1 in PD. Subcutaneous injection of GM1 led to improved UPDRS motor scores versus placebo controls in a 5‐year open study [187]. Additionally, early treatment with GM1 (by 24 weeks) displayed reduced motor disability over 2‐year phase II versus delayed‐start GM1 treatment [186]. The motor impairment normalized at 2‐year phase III follow‐up [186]. These results suggest that increasing GM1 levels has positive outcomes in PD patients. As GM1 does not cross the BBB [188], it may also exert its therapeutic effects on peripheral immune cells or enteric neurons. A GM1 derivative, the GM1 oligosaccharide, also exerts neurotrophic and neuroprotective GM1 functions (reviewed in Chiricozzi *et al*. [65]). GM1 oligosaccharide treatment rescues the neurodegenerative phenotype of *B4galnt1* ^+/−^ mice [189]. Studies are now needed to evaluate whether GM1 oligosaccharide can cross the BBB and has stronger PD modifying properties than GM1.

Glycolipids could also be a relevant therapeutic target for gut inflammation and proteinopathy, which are observed at early stages in PD. Targeting S1P has shown promising results in IBD [190]. Moreover, FTY720 rescues nonmotor symptoms, as shown by the decrease in α‐syn levels in the enteric nervous system and improved gut motility in A53T mice [191]. Potential targets within the GL axis are miRNAs. Indeed, several miRNAs are involved in the regulation of the expression of GL enzymes and receptors [58]. For instance, miRNA‐155 modulates the expression of S1PR_1_ [192, 193] and miRNA profile is altered in PD patients compared with controls [194]. miR‐136‐3p and mir‐433, which are involved in DA synapse, are upregulated in the CSF of PD patients [194]. Other upregulated miRNAs in PD CSF include miR‐153, miR‐409‐3p, miR‐103a, miR‐127‐3p, miR‐10a‐5p, and Let‐7c‐3p while downregulated miRNAs include miR‐1, miR‐22, and miR‐19b‐3p [194]. miRNAs can affect multiple pathways involved in PD. miR‐150, the levels of which are decreased in PD [195], exerts an anti‐inflammatory role. Indeed, LPS‐ and miR‐150‐treated BV2 microglia express lower levels of *Il‐1β*, *Il‐6*, and *Tnfα* genes compared with LPS‐treated BV2 cells [195]. Expression of miR‐7 is significantly reduced in the SN of PD patients [196]. This miRNA can directly regulate *SNCA* expression in miR‐7‐treated HEK293T cells and mice injected with miR‐7‐expressing lentiviral vectors [196]. Lastly, miR‐34b/c levels are decreased in the amygdala, SN, frontal cortex, and cerebellum of PD patients versus controls [197]. Depletion of these miRNAs in SH‐SY5Y cells leads to altered mitochondrial morphology, reduced intracellular ATP levels, and oxidative stress [197]. This list of miRNAs is non‐exhaustive and miRNA levels and function in PD are described in detail in the reviews by Nies *et al*. [198] and Li *et al*. [199]. As microRNAs can be trafficked to cells via exosomes and GLs are exosomal components [58], GLs may indirectly influence cell communication and function in PD by altering exosomal signaling. However, this hypothesis should be tested. Furthermore, whether GLs may directly influence miRNA profile is unknown.

## Conclusions

Glycolipid balance is key to normal body function and its alteration can lead to a variety of diseases involving multiple organs and tissues. GL disturbances can also predispose individuals to develop PD. Increasing evidence suggests that GLs affect cellular functions beyond the brain, including the peripheral immune system, intestinal barrier, and immunity, as well as BBB. GL pathway disturbances are also involved in sporadic PD as well as in aging. Hence, the interplay between aging, genetic predisposition, and environmental exposures could initiate systemic and local GL changes that lead to inflammatory reactions and neuronal dysfunction. We would further argue that targeting the GL axis, especially in selected patients with high polygenic risk profiles, can be an advantageous strategy to prevent or delay the onset of these diseases. Further understanding the cellular and molecular mechanisms that control GL pathways and their impact both in the periphery and in the brain will help unravel how GLs shape the immune and nervous system communication and devise novel drugs to prevent PD and promote healthy aging.

## Conflict of interest

The authors declare no conflict of interest.

## Author contributions

FS and MD wrote the manuscript. FS prepared the figures. MD revised the figures. All authors reviewed the manuscript.

## Data Availability

Data sharing is not applicable to this article as no new data were created or analysed in this study.
